# SNOT-22: psychometric properties and cross-cultural adaptation into the portuguese language spoken in Brazil

**DOI:** 10.5935/1808-8694.20120030

**Published:** 2015-10-20

**Authors:** Guilherme Pilla Caminha, José Tavares de Melo Junior, Claire Hopkins, Emilio Pizzichini, Marcia Margaret Menezes Pizzichini

**Affiliations:** a Otorhinolaryngologist. MSc in Medical Sciences - Federal University of Rio Grande do Sul. PhD in Medical Sciences - Federal University of Rio Grande do Sul; b Otorhinolaryngologist. MSc in Medical Sciences - Federal University of Santa Catarina (UFSC). PhD in Medical Sciences - Federal University of Santa Catarina; c BMBCh FRCS(ORLHNS) DM(Oxon) - Ear, Nose and Throat Department, Guys and St. Thomas' Hospital, London, United Kingdom; d Post-Doctorate in Pneumology; Head of the Pneumology Department - UFSC. Associate Professor of Internal Medicine - UFSC. Coordinator of NUPAIVA/UFSC, Member of GINA's Scientific Committee; e Post-Doctorate in Pneumology; Associate Professor - Department of Internal Medicine at UFSC and Coordinator of the Graduate Program in Medical Sciences - UFSC. Chair of the Asthma Committee - SBPT

**Keywords:** quality of life, questionnaires, sinusitis

## Abstract

Rhinosinusitis is a highly prevalent disease and a major cause of high medical costs. It has been proven to have an impact on the quality of life through generic health-related quality of life assessments. However, generic instruments may not be able to factor in the effects of interventions and treatments. SNOT-22 is a major disease-specific instrument to assess quality of life for patients with rhinosinusitis. Nevertheless, there is still no validated SNOT-22 version in our country.

**Objective:**

Cross-cultural adaptation of the SNOT-22 into Brazilian Portuguese and assessment of its psychometric properties.

**Method:**

The Brazilian version of the SNOT-22 was developed according to international guidelines and was broken down into nine stages: 1) Preparation 2) Translation 3) Reconciliation 4) Backtranslation 5) Comparison 6) Evaluation by the author of the SNOT-22 7) Revision by committee of experts 8) Cognitive debriefing 9) Final version. Second phase: prospective study consisting of a verification of the psychometric properties, by analyzing internal consistency and test-retest reliability.

**Results:**

Cultural adaptation showed adequate understanding, acceptability and psychometric properties.

**Conclusion:**

We followed the recommended steps for the cultural adaptation of the SNOT-22 into Portuguese language, producing a tool for the assessment of patients with sinonasal disorders of clinical importance and for scientific studies.

## INTRODUCTION

Rhinosinusitis is a problem that reaches surprising dimensions. Approximately 15% of the population in industrialized countries has nasal or paranasal problems, making it the second most prevalent condition among chronic diseases,^1^ with an annual socio-economical cost estimated in 6 billion USD in the USA^2^. Although rarely causing admissions to the emergency room, an almost always non-fatal, chronic nasosinusal disease have a substantial negative impact on the health and quality of life of affected individuals, adversely affecting mood, social and physical performance.

Such impact has been proven using global measures of quality of life, such as the SF-36 (*Short Form 36 Health Survey Questionnaire*)^3^, showing that rhinosinusitis has more consequences on physical pain and social performance than angina, congestive heart failure, back pain and chronic obstructive pulmonary disease^4^. If on the one hand these global scores help compare it to other chronic diseases, on the other hand it does not assess the most important specific aspects associated with the disease^5^. For instance, the most generic instruments may not be capable to factor in the effects of interventions and treatment. The development of disease-specific questionnaires has filled out this gap in the assessment of patients with nasosinusal diseases. Today, there are numerous specific questionnaires used to analyze the quality of life of patients with rhinosinusitis^6^ and the SNOT-22 (*22 item Sinonasal Outcome Test*)^7^ is a SNOT-20 modified questionnaire^8^, from which the importance score was removed and two other items were added: nasal obstruction and the loss of olfaction and taste. It is an instrument broadly used in the assessment of patients with nasosinusal diseases, bearing transcultural adaptations to other languages. The goal of the present paper was to culturally adapt and validate the SNOT-22 questionnaire for Brazilian Portuguese.

## METHOD

This study was carried out in two stages: a) the SNOT-22 questionnaire adaptation into the Portuguese language spoken in Brazil and, b) a prospective study to analyze the psychometric properties by means of internal consistence and test-retest reliability. The study was approved by the Ethics in Research with Human Beings Committee of our institution, certified under n^o^ 582 and carried out according to ethical principles.

### Cultural adaptation

The same process employed in the cultural adaptation of a questionnaire can be found in another publica-tion^9^. Following, we will summarize the steps used in the cultural adaptation: 1) Preparation: getting the author's permit to translate and culturally adapt the questionnaire. 2) Translation of three versions from English into Portuguese by three physicians, independently. 3) Reconciling among the translators and creation of one single version into Portuguese. 4) Translation of the single version in Portuguese into English, made by a native speaker of English, also fluent in Portuguese. 5) Comparison of this latest translation into English with the original SNOT-22 version in English in order to look for possible differences. 6) Assessment and approval of the SNOT-22 retranslated into English from the final Portuguese version by the author. 7) Review of the SNOT-22 Portuguese version by a committee of specialists made up of two otorhinolaryngologists and two pneumologists. 8) Cognitive unfoldings 1 and 2: the cognitive unfolding is the process of testing the understanding of the translated questionnaire by the target population. Thus, 20 patients were interviewed at each stage of the unfolding - 40 patients in total - all with sinonasal complaints and who had good understanding and language. The questionnaire was applied by the authors to each participant, and following, they were asked about how much they understood each item. A form concerning their understanding of each item was scored between 1 and 10. It was conceived that scores between 1 and 4 would indicate a confusing statement, between 5 and 7 a not so clear statement, and between 8 and 10, a clear statement^10^. The level of clarity was obtained by the mean value of adding the scores assigned by the patients. The statements which did not reach 0.4 had to be replaced, the statements which did not reach 0.8 should be rephrased and, finally, those which had a final index equal to or higher than 0.8 were considered adequate as far as their understanding was concerned. 9) Final version: the goal of this stage was to produce the final version of the instrument adapted for the Portuguese language spoken in Brazil. All the participants of the previous stages, except for the patients, were gathered in order to produce the final version of the process of cultural adaptation and translation of the SNOT-22 for the Portuguese language spoken in Brazil. At this stage, the instrument was revised item by item, in which we discussed the findings from the cognitive unfoldings, incorporating the pertaining changes and creating the final version of the symptom-specific questionnaire to assess the quality of life of patients with chronic rhinosinusitis.

### Psychometric properties

The assessment of the psychometric properties in this instrument was carried out by means of analyzing the internal consistency and temporal stability, both are reliability indicators. The assessment of internal consistency in Portuguese of the SNOT-22 was carried out by means of the Cronbach's alpha coefficient, considering 0.70 as the acceptable value for the lower limit. The test-retest reliability was checked by the interclass correlation coefficient (ICC) in 16 patients with nasosinusal complaints with stable disease approximately seven days after the first assessment. The sample size for the test and retest was estimated according to the ICC accuracy calculation. Thus, the size was calculated according to Bonett^11^ and based on the estimate of the questionnaire being filled out by the same individual in two different occasions, hoping to find an ICC of 0.9 and an alpha value of 0.05. With these assumptions, a sample of 16 patients is more than adequate. For this type of study, an ICC greater than or equal to 0.8 is an indicator of good reliability. And finally, the test's reproducibility was investigated thoroughly based on the graphic tool proposed by Bland & Altman^12^, in which the difference between the first and the second scores (Y axis) is represented by the mean value between the first and the second scores (X axis). This methodology enables a better perception of the reproducibility of values between the patients, as well as assesses the distribution range of the values within the 95% confidence interval (mean ± two standard deviations).

## RESULTS

**Figure 1:**
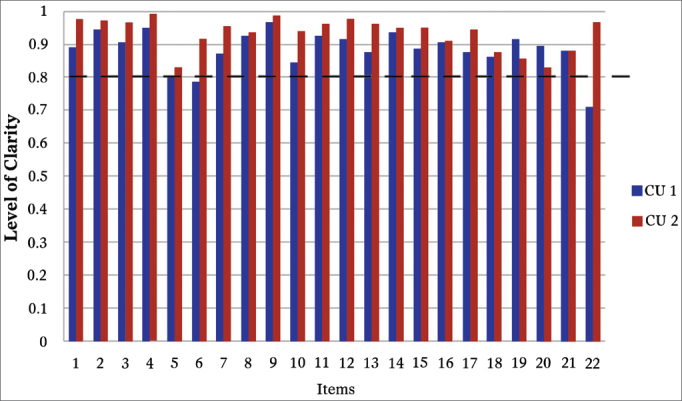
Establishing the clarity of SNOT-22. This shows the clarity index from each item in the “SNOT-22”. The blue bars represent the clarity index upon the first cognitive unfolding and the red bars indicate the clarity index upon the second cognitive unfolding. The dotted line shows the cutting point of 0.80 from which each item is considered clear. We notice that items 6 and 22 are located below the clarity index upon the first stage of unfolding and that all the items in “SNOT-22” were considered clear in the second cognitive unfolding. CU: cognitive unfolding.

Throughout the SNOT-22 reconciling stage, the review committee discussed, standardized and uniformed the terms which became part of version 1 in Portuguese. Its back-translation was fully accepted by the author of the original SNOT-22; therefore, without the need to change the first version. The Portuguese language review stage for SNOT-22 by the expert committee was branded by numerous changes, which will be detailed as follows: in the questionnaire's statement, the term “two weeks” was written in bold type, so as to stress the importance of the assessment period; moreover, in the explanatory chart, there was a change in the text structure, aiming at improving its understanding. Thus, the following phrase: “*Considering how severe the problem is when you noticed it and the frequency at which it happens, please quantify each of the items below, circulating the number corresponding to “how bad” you feel. Use the scale on the side” has been changed to*: “*Look at the symptoms listed below, numbered from 1 to 22. Following, use the scale next to it to assess the severity of your problem and how often it happens. To end, circle the number corresponding to how bad you feel*”; on the gradation scale, the last item corresponds to a score of 5, which said: “*Problem as bad as possible*” has been substituted for “*Very severe problem*”; item 5, which had the phrase: “Post-nasal dripping *(a feeling of secretion or sputum running down the back of your nose)*”, has been substituted for “A feeling of secretion or sputum running down the back of your nose”, taking out the complex term “*post-nasal dripping*” and adding the noun “sputum” for emphasis purposes. Item 6, which had the following phrase: “Thick nasal secretion (thick mucus in the nose)”, was substituted for “*Thick mucus or sputum in the nose*”; item 10, which had the phrase: “*Facial pain or pressure*”, was substituted for: “*Pain or pressure on the face*”; item 12, which had the phrase: “Wake up at night”, was rephrased for “*Waking up in the middle of the night*”; item 14, which had the phrase: “*Waking up tired*”, was added the term “*in the morning*”; also to item 15, which had the phrase: “*Tiredness/fatigue*” the following complement was added: “*Tiredness/fatigue throughout the day*”; to item 16, the following explanatory term was added: “*Reduced productivity (lower performance)*”; by the same token, to item 21, which had the phrase: “*Perception of olfaction or taste*” an explanatory term was added, as follows: “*Perception of olfaction (smell) or taste*”. Thus, this stage peaked at producing the second version of the SNOT-22 translated into Portuguese. In the stage of the first cognitive unfolding, two items from the SNOT-22 were difficult to understand (Figure 1). Item number 6, had a clarity index of 0.785. Consequently, the phrase “*Thick mucus or sputum in the nose*” was changed to “*Thick sputum in the nose (thick mucus in the nose)*”. Item number 22, which reached the clarity index of 0.71, required the substitution of the term “*Nasal obstruction/congestion*” for “Locked/Stuffed nose”. Thus, a third version of the SNOT-22 was created for Portuguese, with the corrections and adaptations mentioned above. And finally, on the second stage of cognitive unfolding, questions 6 and 22, which had been modified in order to optimize the clarity index, reached, at the time, indices of 0.915 and 0.965, respectively. The remaining indices were all kept above 0.80, thus making it unnecessary to unfold again (Figure 1).

And finally, the reconciling stage resulted in the Final Version, which content embodied all the aforementioned changes (Chart 1).

**Chart 1:** Final Version of the SNOT-22 Questionnaire. SNOT-22 Sinonasal Outcomes Questionnaire.

Name:______________________Date:__/__/___						
Below, there is a list of symptoms and social/emotional consequences associated with your nasal problem. We would like to know more about these problems, and we appreciate your time in answering these questions to the best of your abilities. There are no correct or wrong answers, and only you can provide us with this information. Please quantify your problems and how they have presented in the last **two weeks**. Thank you for your participation.						
A: Please, read the symptoms below, numbered from 1 to 22. Following, use the scale to the side to quantify the severity of your problem and the frequency at which it happens. To end, please circle the number corresponding to how bad you feel →	No problem	Very Mild Problem	Mild or light problem	Moderate problem	Severe problem	Very severe problem
1. Need to blow your nose	0	1	2	3	4	5
2. Sneezing	0	1	2	3	4	5
3. Running nose	0	1	2	3	4	5
4. Cough	0	1	2	3	4	5
5. A feeling of secretion running down the back of your nose	0	1	2	3	4	5
6. Thick secretion in the nose (thick mucous in the nose)	0	1	2	3	4	5
7. Stuffed ear (clogged ear)	0	1	2	3	4	5
8. Dizziness	0	1	2	3	4	5
9. Ear ache	0	1	2	3	4	5
10. Facial pain or pressure	0	1	2	3	4	5
11. Difficulty falling asleep	0	1	2	3	4	5
12. Waking up in the middle of the night	0	1	2	3	4	5
13. Lack of a good night of sleep	0	1	2	3	4	5
14. Wake up tired in the morning	0	1	2	3	4	5
15. Tiredness/fatigue throughout the day	0	1	2	3	4	5
16. Reduced productivity (lower performance)	0	1	2	3	4	5
17. Reduced concentration	0	1	2	3	4	5
18. Frustrated/impatient/touchy	0	1	2	3	4	5
19. Sad	0	1	2	3	4	5
20. Embarrassed	0	1	2	3	4	5
21. Perception of olfaction (smell) or taste	0	1	2	3	4	5
22. Clogged/stuffed nose	0	1	2	3	4	5
Total		____	____	____	____	____
					Total General_______	

### Psychometric Properties

**Figure 2:**
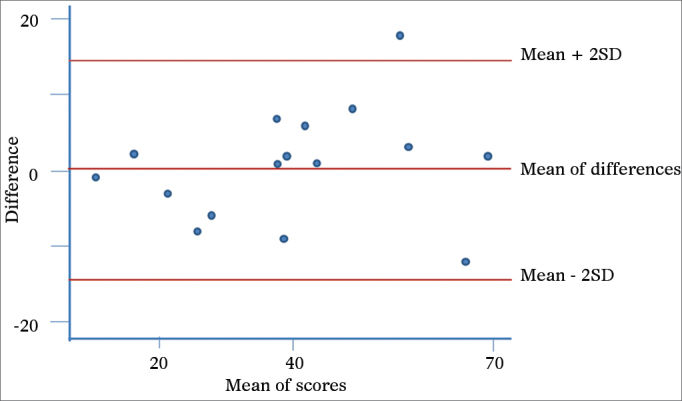
Graphical representation as proposed by Bland and Altman – reproducibility. Scores from the first and second visit. On the Y axis, the difference between the scores from the first and second administrations of the questionnaire and, on the X axis, their mean values. The central red line indicates the difference between the scores from the first and second administrations of the questionnaire and the peripheral red lines indicate the mean ± two standard deviations (1.96 × SD). SD: Standard Deviation.

The instrument's final version presented the Cron-bach alpha coefficient of 0.88, showing good instrument internal consistency, and an intraclass correlation coefficient of 0.91, indicating good reliability. We also noticed that most of the times the differences in scores between the first and the second assessments were within the levels of agreement of 95% [mean ± (1.96 × standard deviation)]. Moreover, there was no association between the differences of the scores and their mean values (Figure 2).

## DISCUSSION

The use of disease-specific questionnaires has been adding valuable information to scientific knowledge. Based on a robust and internationally accepted methodology, we have culturally adapted the SNOT-22 questionnaire to the Portuguese Language spoken in Brazil. The methodology employed confirmed its reliability and reproducibility, assuring that it can be used in the clinical setting as well as in research.

Today, there are specific and validated instruments used to assess sinonasal symptoms, and the choice of one in particular must be guided by the goals to be attained. Knowingly, for such an instrument to be clinically useful, it must be validated and adequate for the disease at hand, reliable and responsive to changes, and easy to be interpreted and filled out^13^. Here, the SNOT-22 deserves special attention, not only for being easy to fill out and understand, but also because of its properties which have been already validated in numerous studies^7^, ^14^, ^15^, ^16^. Notwithstanding, since it is an instrument which was originally created in English, it is inadequate - if not impossible - to use from a literal translation.

The cultural adaptation of a questionnaire is a rather complex process which involves a translation conceptually equivalent to the original document, and culturally acceptable in the country where the questionnaire will be used. Therefore, it is paramount to search for technical and semantical equivalence between the original version and the adapted one in order to prevent distortions in this process which can cause changes to the psychometric properties of the instrument at hand^17^. In the SNOT-22 translation and cultural adaptation, our study was carried out according to the instructions in the literature^18^, ^19^ and, in particular, the guidelines from the *International Society for Pharmacoeconomics and Outcomes* (ISPOR)^20^ thus, pursuing the semantical and conceptual equivalences between the original instrument and its Portuguese version in the context of the Brazilian culture. Upon assuring such equivalence, we expect to maintain the psychometric properties of SNOT-22, which have been properly documented in prior studies.

Moreover, we understand it is of paramount importance to have the participation of the author of the original questionnaire in the stages of appreciation of the retranslated construct so as to guarantee the accuracy of the translated version. In addition, in this study we reassessed the psychometric properties as to internal consistency (Cronbach's index = 0.88) and the test-retest reliability (ICC = 0.91), showing - similarly to previous studies - proper internal consistency and reliability. By using the graphical representation proposed by Bland & Altman, we showed that most of the differences were located between the agreement thresholds. Moreover, there was no association between the differences and the mean, in other words, the severity and the impact of the problem detected had no effect on the temporal stability. We noticed that there was an *outlier*, which the difference between the first and the second scores was of 18 points. According to prior studies, the minimally important difference, defined as the lowest change in the score which may be perceived by the patient is 8.9. Such situation may represent more than the learning effect vis-à -vis the instrument, a true worsening of the patient's clinical conditions.

Concerning the assessment of reliability, we know that, by definition, the SNOT-22, is a self-applicable questionnaire. Thus, all the interviews were carried out by researchers; however, the complete reading of the items, as well as filling out the answers was tasks carried out only by the patients, without any interference from the researchers. We should stress that Kosugi et al.^21^, upon carrying out the first stage of the reliability check (test), read the items for the patients. In such cases we see a distance from the cultural adaptation object, since the original instrument had its conception and check of psychometric properties, both being structures based upon self-administration. In other words, it is not adequate to adapt an instrument which was originally conceived to be self-administered into a format at which the interviewer plays the role of conveying information. On the second stage (retest), the same group made the interviews by phone. It is know that the patients may answer differently to an instrument when it is provided on paper or in electronic means, which may compromise again the reliability check of the instrument^22^. In a general context, we understand there has been damage to the internal validity when the instrument is employed by third parties and through the telephone, since these bring about potential loss in accuracy. And finally, the external validity was compromised, considering that such methodology does not guarantee that this instrument be truly understandable by the target public through its self-administration, or that the results be equivalent to those of other studies, especially concerning responsiveness and the clinically important minimum difference.

Concerning its use, this is an instrument that is easy to answer, in a short time and it may be employed prior to the consultation. Moreover, it enables to systematically obtain the key-points in the disease history and educates the patient, since it invites the patient to pay attention to the most common signs and symptoms of the disease, as well as its severity. These benefits may result in earlier treatments and help avoid the risk of complications. Thus, the instrument will assess different aspects of the clinical expression of chronic rhinosinusitis from different etiologies, considering the different symptoms, avoiding a specific set of questions for each one of the multiple manifestations of the disease by the physician and enabling better care of patients with rhinosinusitis.

As to the SNOT-22 limitations, in general, it is known that life quality measures impose standardized domains which were built based on the observations of the entire population. As a result, we may be restricting the individual choices of the patients and ultimately influencing the capacity of the instrument to respond to changes after treatment. In the specific realm of cultural adaptation, we know Brazil is a large country with different geographic and population characteristics - yielding a very peculiar cultural diversity. Moreover, the social discrepancies bring about gaps in the educational background of Brazilians. Attentive to it, we took the care of using easy-to-understand words, which we believe to be understandable in the entire national territory.

## CONCLUSION

The SNOT-22 cultural adaptation to the Portuguese language spoken in Brazil brings to our language and culture a self-applicable instrument that has been internationally accepted and which preserves the validity of the original questionnaire. This is an important tool to use both in research as well as in clinical practice, to assess the impact sinonasal disease of different etiologies have in patients' lives. In addition, its psychometric properties (responsiveness and clinically important minimum differences) may be valuable in assessing the effects of different interventions during the course of the disease and to discriminate its impact on the quality of life of different patient subgroups.
